# H19/let-7/LIN28 reciprocal negative regulatory circuit promotes breast cancer stem cell maintenance

**DOI:** 10.1038/cddis.2016.438

**Published:** 2017-01-19

**Authors:** Fei Peng, Ting-Ting Li, Kai-Li Wang, Guo-Qing Xiao, Ju-Hong Wang, Hai-Dong Zhao, Zhi-Jie Kang, Wen-Jun Fan, Li-Li Zhu, Mei Li, Bai Cui, Fei-Meng Zheng, Hong-Jiang Wang, Eric W-F Lam, Bo Wang, Jie Xu, Quentin Liu

**Affiliations:** 1 Institute of Cancer Stem Cell, Dalian Medical University, Dalian; State Key Laboratory of Oncology in South China, Cancer Center, Sun Yat-sen University, Guangzhou 510060, China; 2 Department of Breast Surgery, The Second Affiliated Hospital of Dalian Medical University, Dalian 116023, China; 3 Department of Oncology, The First Affiliated Hospital of Dalian Medical University, Dalian 116011, China; 4 Department of Hematology, The Second Affiliated Hospital of Dalian Medical University, Dalian 116023, China; 5 Department of Obstetrics and Gynaecology, The First Affiliated Hospital of Dalian Medical University, Dalian 116011, China; 6 Department of Medical Oncology, The Eastern Hospital of The First Affiliated Hospital, Sun Yat-Sen University, Guangzhou 510700, China; 7 Department of Breast Surgery, The First Affiliated Hospital, Dalian Medical University, Dalian 116011, China; 8 Department of Surgery and Cancer, Imperial College London, London W12 0NN, UK

## Abstract

Long noncoding RNA-H19 (*H19*), an imprinted oncofetal gene, has a central role in carcinogenesis. Hitherto, the mechanism by which H19 regulates cancer stem cells, remains elusive. Here we show that breast cancer stem cells (BCSCs) express high levels of *H19*, and ectopic overexpression of *H19* significantly promotes breast cancer cell clonogenicity, migration and mammosphere-forming ability. Conversely, silencing of *H19* represses these BCSC properties. In concordance, knockdown of *H19* markedly inhibits tumor growth and suppresses tumorigenesis in nude mice. Mechanistically, we found that H19 functions as a competing endogenous RNA to sponge miRNA let-7, leading to an increase in expression of a let-7 target, the core pluripotency factor LIN28, which is enriched in BCSC populations and breast patient samples. Intriguingly, this gain of LIN28 expression can also feedback to reverse the H19 loss-mediated suppression of BCSC properties. Our data also reveal that LIN28 blocks mature let-7 production and, thereby, de-represses H19 expression in breast cancer cells. Appropriately, *H19* and *LIN28* expression exhibits strong correlations in primary breast carcinomas. Collectively, these findings reveal that lncRNA H19, miRNA let-7 and transcriptional factor LIN28 form a double-negative feedback loop, which has a critical role in the maintenance of BCSCs. Consequently, disrupting this pathway provides a novel therapeutic strategy for breast cancer.

Breast cancer is the leading cause of cancer-related death among females worldwide.^1, 2^ Although early diagnosis and more effective treatment strategies have diminished the mortality rates in recent years,^3, 4, 5^ the development of recurrence, metastasis and chemoresistance is, in most cases, still inevitable.^6^ Breast cancer stem cells (BCSCs) have been shown to exhibit unique characteristics, including enhanced self-renewal, dedifferentiation and resistance to chemotherapy or radiotherapy, all of which are believed to contribute to the development and overall aggressiveness of the recurrent or metastatic lesions.^7, 8, 9^ The initial evidence for the existence of cancer stem cells (CSCs) in breast cancer reveals that only the minority of breast cancer cells with a CD44^+^/CD24^−/low^/ESA^+^ phenotype have the ability to form new tumors in NOD/SCID mice.^10^ In addition, the CD44^+^/CD24^−^ phenotype is enriched in basal-like breast tumors^11^ and related to triple-negative state in breast cancer.^12^ BCSCs also present high aldehyde dehydrogenase 1 (*ALDH1*) expression and enhance ‘side population' (SP) cells that efflux the Hoechst dye via ABCG2 (a key factor contributing to drug resistance in cancers).^13, 14^ Furthermore, BCSCs possess the property to form mammospheres, which are thought to be enriched mammary stem cells.^15^ Therefore, eliminating BCSCs provide a therapeutic avenue for overcoming chemoresistance, metastasis and relapse in breast cancer patients.^16^

Long noncoding RNA *H19* is maternally imprinted and locates close to the telomeric region of chromosome 11p15.5.^17^ This gene is activated in murine extraembryonic cell types at the time of implantation, but is subsequently expressed in all of the mid-gestation embryo cells excluding most of the developing central and peripheral nervous systems. After birth, the expression of this gene ceases or markedly decreases in all tissues.^18^ Maternal allele-specific deletion of the *H19* differential methylated region maintains hematopoietic stem cell repopulating ability through a *miR-675-Igf1* signaling circuit.^19^ A critical *trans*-regulatory function in skeletal muscle differentiation and regeneration is mediated by the microRNAs (miR-675-3p and miR-675-5p) encoded within *H19*.^20^ Moreover, H19 serves as a molecular sponge to regulate the bioavailability of tumor suppressor miRNA let-7 during the process of endometrial cancer metastasis.^21^ Although recent findings have indicated that H19 has important roles in regulating tumorigenicity^22^ and stemness of glioblastoma,^23^ little is known about the mechanism by which H19 controls cancer stem cell maintenance.

A highly conserved RNA-binding protein LIN28 is a member of reprogramming factors acted in concert with *KLF4*, *SOX2* and *NANOG*, to induce pluripotency in adult human fibroblast cells.^24, 25^ Accumulating evidence has indicated that LIN28 is overexpressed in advanced human malignancies and has a key role in the maintenance of CSCs.^26, 27^ LIN28A binds to conserved terminal loop of pre-let-7 elements and induce terminal uridylation (addition of uridine nucleotides) of pre-let-7 via recruitment of Zcchc11, a terminal uridylyl transferase (TUTase) and TUT4.^28^ After that, DICER is unable to cleave uridylated pre-let-7 transcript, inhibiting the production of mature let-7 miRNAs (a key ‘keeper' of the differentiated state of embryonic stem cells).^29^ Thus, blockage of let-7 production and subsequent de-repression of let-7 miRNA target genes (*RAS*,*MYC* and*HMGA2*) by LIN28 has an essential function in CSC maintenance.^30, 31^ Moreover, LIN28 post-transcriptionally upregulates *LGR5* and *PROM1* through let-7-independent mechanism, promoting colon cancer progression and metastasis.^26^

Here we demonstrate that *H19* expression is enriched in BCSC subpopulations and breast tumor samples. As a competing endogenous RNA (ceRNA), H19 increases LIN28 expression by blocking the bioactivity of let-7, an upstream repressor of *H19*. In addition, LIN28 induction can further reduce let-7 expression in a feedback mechanism. Taken together, our results suggest that H19/let-7/LIN28 forms a double-negative reciprocal circuitry to facilitate BCSC maintenance.

## Results

### H19 is highly expressed in human breast tumors and BCSC subpopulations

**Figure 1:**
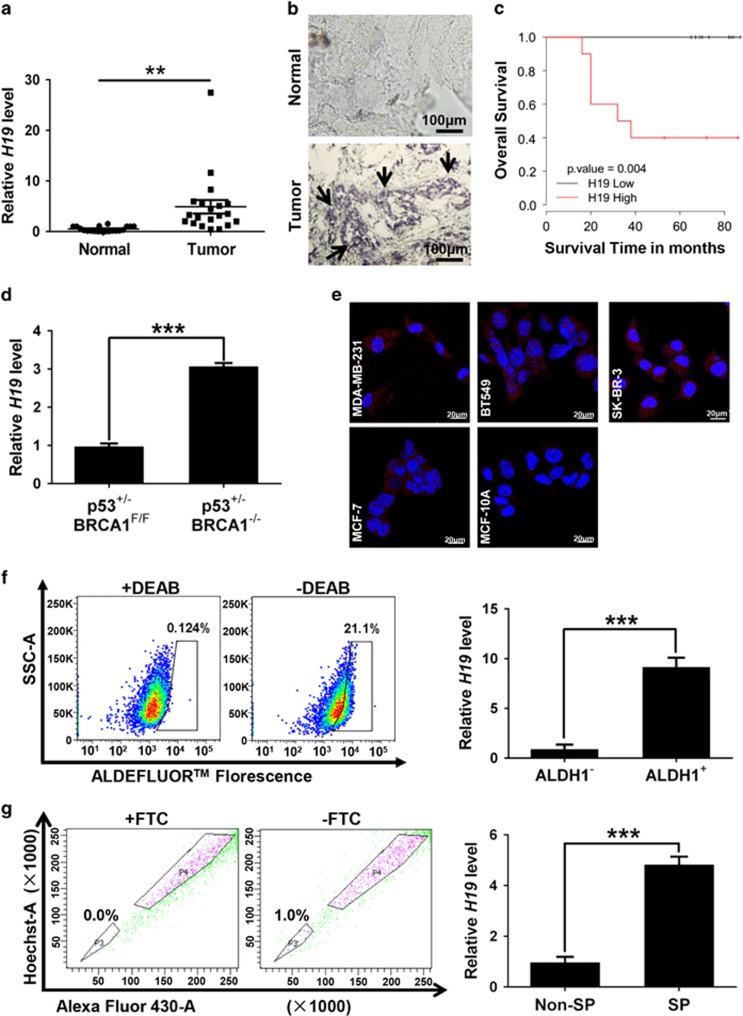
LncRNA-*H19* expression is assessed in clinical breast cancer specimens and cancer cell lines. (**a**) Analysis of *H19* expression in breast cancerous tissues and adjacent normal tissues (*n*=20). The relative *H19* mRNA level was normalized to *ACTB*. The statistical differences were analyzed using the paired *t*-test. (**b**) *In situ* analysis with a DIG-labeled H19 probe in breast cancerous tissue and adjacent normal tissues. The scale bar represents 100 *μ*m. (**c**) Kaplan–Meier survival analysis was performed to investigate the implication of H19 level on patient overall survival (*n*=20). (**d**) Mice harboring *BLG-Cre*; *Brca1*^*F*22–24*/F*22–24^; *p53* ^+*/*−^ were established by two rounds of pregnancy and then tumors developed were collected (*n*=3). H19 expression was further verified in tumor and mammary tissues by RT-qPCR assay. (**e**) The *in situ* expression of *H19* RNA (red) was detected by FISH assay. The red fluorescent signal is from the *H19* RNA probe, and the blue fluorescent signal is from nuclear DNA counterstained with DAPI. The scale bar represents 20 *μ*m. (**f**and **g**) Detections of *H19* expression in the ALDH1-positive (ALDH1^+^) subpopulation (**f**) and side population (SP) cells (**g**) are showed by dot plots by FACS and the relative expression levels in MDA-MB-231 cells. Data are represented as mean±S.D. **P<0.01 and ***P<0.001, *n*=3

To investigate the relationship between H19 and BCSCs, we assessed the expression of *H19* in breast cancer tissues and breast cancer cells. In breast cancer samples, *H19* levels were significantly higher in cancerous tissues compared with the adjacent normal tissues (Figure 1a, *P*<0.01; Figure 1b). In addition, the Kaplan–Meier survival analysis demonstrated that high *H19* level was a strong indicator for an inferior overall survival in breast cancer patient samples (Figure 1c). Furthermore, breast tumors were induced in mice (*Brca1*^*−/−*^ *p53* ^+*/*−^), and RT-qPCR assay showed that H19 was significantly upregulated in tumors compared with normal mammary glands (Figure 1d). Consistently, *H19* expression was detected at higher levels in breast cancer cells than in breast epithelial cells and stemness-related factors OCT4, SOX2 and NANOG were expressed at higher levels in MDA-MB-231 cells compared with MCF-10A cells (Figure 1e and Supplementary Figure 1A). We next sorted ALDH1^+^ (Figure 1f, left) and SP cells (Figure 1g, left) from MDA-MB-231 cells by fluorescence-activated cell sorting (FACS) to enrich BCSC subpopulations. Notably, the expression of *H19* was significantly enhanced in ALDH1^+^ (Figure 1f, right, *P*<0.001) and SP cells (Figure 1g, right, *P*<0.001). *POU5F1*,*SOX2* and *NANOG* were highly expressed in BCSC-enriched populations compared with non-enriched cells (Supplementary Figure 1B). Moreover, the expression of *H19* was also elevated in sphere-forming (Supplementary Figure 1C) and three-dimensional (3D) culture (Supplementary Figure 1E) cells. Similarly, the expression of *POU5F1*, *SOX2* and *NANOG* in sphere-forming (Supplementary Figure 1D) and 3D culture (Supplementary Figure 1F) cells were increased to determine the BCSC enrichment. These results demonstrate that BCSCs express higher levels of *H19*.

### H19 is required for the maintenance of BCSC characteristics

**Figure 2:**
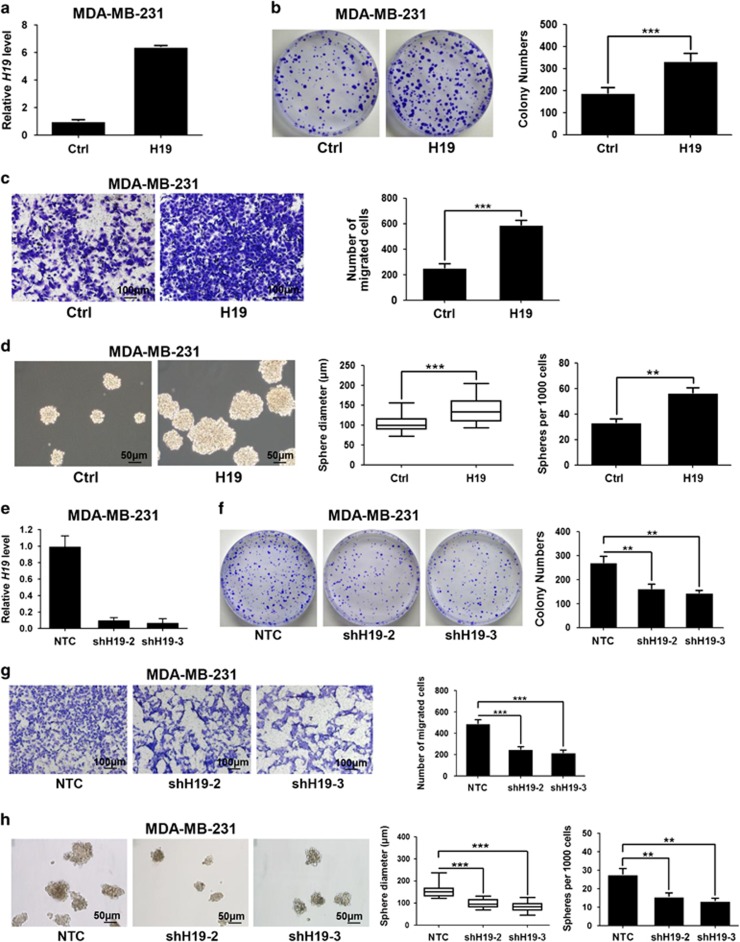
LncRNA-H19 is involved in the maintenance of CSC characteristics in breast cancer cells. (**a**) Ectopic expression of *H19* mRNA expression was confirmed by RT-qPCR after lentivirus infection in MDA-MB-231 cells. (**b**) Ectopic overexpression of *H19* enhanced clonogenic growth *in vitro*. Representative images of colonies were presented (left) and colony numbers were counted after culture for 12 days (right). Data are represented as mean±S.D. ***P<0.001, *n*=3. (**c**) Cell migration was analyzed in *H19* overexpression and control groups. Stained images of invaded cells are presented (left) and migrated numbers are mean±S.D. (*n*=3), ***P<0.001 (right). (**d**) Mammosphere formation was increased by *H19* overexpression. Representative images were presented (left), and the size and numbers of mammospheres were counted (right). Data were shown as mean±S.D. from three independent experiments, **P<0.01 and ***P<0.001, respectively. (**e**) The interfering efficiency of the lentivirus encoding *H19*-targeting shRNAs (shH19) were confirmed by RT-qPCR, compared with negative control lentivirus (NTC). (**f**–**h**) Knockdown of *H19* reduced breast cancer cells clonogenicity (**f**), migration (**g**) and mammosphere-forming ability (**h**). Data were shown as mean±S.D. from three independent experiments, **P<0.01 and ***P<0.001, respectively

To assess the role of *H19* in the regulation of BCSCs properties, we established stable *H19*-overexpressing MDA-MB-231 cells and confirmed the enforced expression of *H19* by RT-qPCR (Figure 2a). Then, we performed colony formation, transwell migration and sphere formation assays. Colony numbers were distinctly increased in *H19-*overexpressing MDA-MB-231 cells compared with controls (Figure 2b, *P*<0.001). Number of migration cells was also markedly elevated in *H19*-overexpressing cells (Figure 2c, *P*<0.001). In addition, sphere formation capacity was significantly enhanced upon *H19* overexpression (Figure 2d, left). Both size and number of spheres were markedly elevated in *H19*-overexpressing cells (Figure 2d, middle, *P*<0.001; Figure 2d, right, *P*<0.01). Moreover, overexpressing *H19* in another breast cancer cell line SK-BR-3 (Supplementary Figure 2A), also resulted in similar increases in self-renewal properties (Supplementary Figures 2B–D). In contrast, when *H19* was knocked down by short hairpin RNAs (shRNAs) in MDA-MB-231 and SK-BR-3 cells (Figure 2e; Supplementary Figure 2E), there was a significant reduction in clonogenicity, migration and sphere-forming ability in MDA-MB-231 (Figures 2f–h) and SK-BR-3 cells (Supplementary Figures 2F–H). Interestingly, overexpression or knockdown of *H19* displayed no effects on cell proliferation in MDA-MB-231 (Supplementary Figures 3A and B) and SK-BR-3 cells (Supplementary Figures 3C and D). Together, these data showed that H19 is crucial for the maintenance of BCSC properties *in vitro.*

### H19 is essential for tumorigenesis and tumor growth *in vivo*

**Figure 3:**
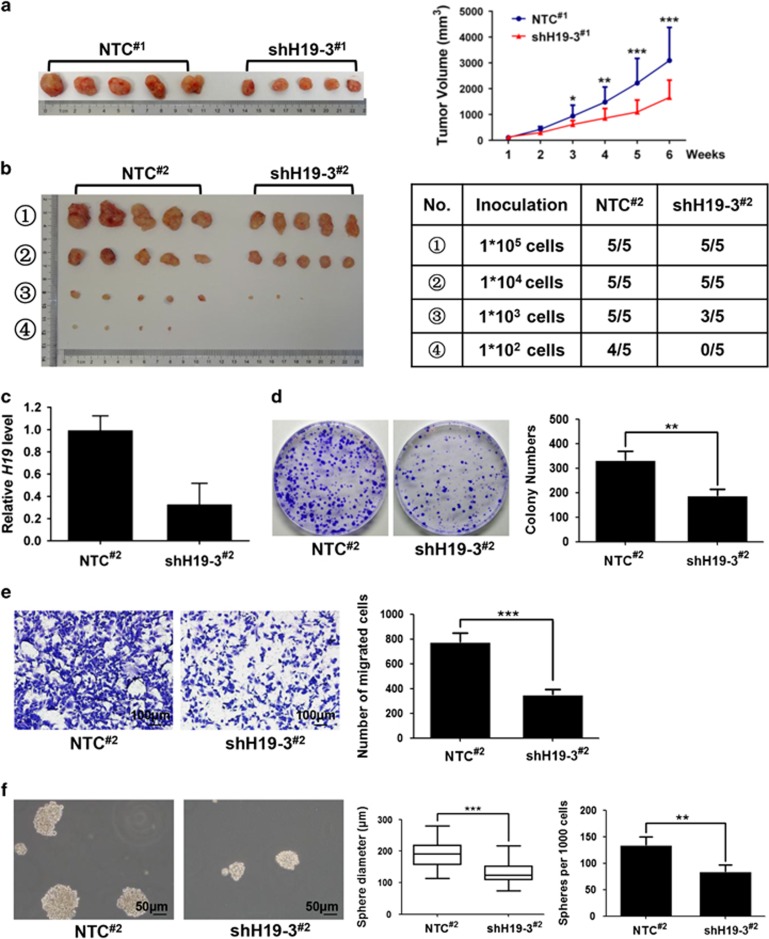
Endogenous LncRNA-H19 is required for CSCs maintenance *in vivo*. (**a**) *H19* depletion attenuated xenograft tumor growth after the first tumor transplantation. MDA-MB-231 cells were infected with lentivirus shH19-3 or NTC; cells were subcutaneously injected into nude mice, respectively. Representative images of subcutaneous tumors taken 6 weeks post inoculation (left) and the tumor growth curves were presented (right). Data are presented as the mean ± S.D. (*n* = 5; *P<0.05, **P<0.01 and ***P<0.001). (**b**) *H19* knockdown significantly repressed *in vivo* tumor formation after second serial tumor transplantation. Shown were representative images of subcutaneous tumors collected at end point (left) and summary of tumor xenografts formation of different groups in nude mice (right). (**c**) The knockdown efficiency of *H19* in the second tumor xenografts were detected by RT-qRCR. Error bars represent mean±S.D. of triplicates. (**d**–**f**) The cancer cells isolated from *H19* knockdown tumor xenografts displayed the reduced clonogenicity (**d**), migration (**e**) and mammosphere-forming (**f**) abilities. Data were shown as mean±S.D. from three independent experiments, **P<0.01 and ***P<0.001, respectively

As continuous tumor growth could be sustained by BCSCs, we performed serial transplantation in nude mice to investigate the *in vivo* role of H19 in the regulation of BCSC maintenance. In the first tumor transplantation, either MDA-MB-231-shCtrl (NTC) or MDA-MB-231-shH19 (shH19) cells were subcutaneously transplanted into nude mice (*n*=5). As shown in Figure 3a, the mice injected with shH19 cells formed apparently smaller tumor mass than the mice injected with NTC cells, indicating that H19 was critical for tumor growth. Importantly, the cells isolated from shH19 tumor xenografts, with a 77.6% knockdown efficiency (Supplementary Figure 4A), displayed diminished clonogenicity, migration and sphere-forming ability compared with the cells from NTC tumor xenografts (Supplementary Figures 4B–D). Furthermore, *H19* depletion significantly repressed the second limited dilution tumor transplantation. As shown in Figure 3b, not only the tumor volumes but also the tumor formation efficiencies decreased in the shH19 tumor xenografts compared with the NTC tumor xenografts. When shH19 cells (1 × 10^2^) were injected into nude mice (*n*=5), these cells failed to generate an observable tumor mass. In contrast, the same number of NTC cells generated tumor mass in four out of five mice. Furthermore, the clonogenicity, migration and sphere-forming ability remained substantially lower in the cells isolated from the shH19 tumor xenografts of the second transplantation, with 66.8% knockdown efficiency of *H19* (Figure 3c) in comparison with the cells from the NTC tumor xenografts (Figures 3d–f). These data strongly supported that H19 has a critical role in the maintenance of BCSCs *in vivo*.

### H19 functions as a molecular sponge for let-7 miRNA in breast cancer cells

**Figure 4:**
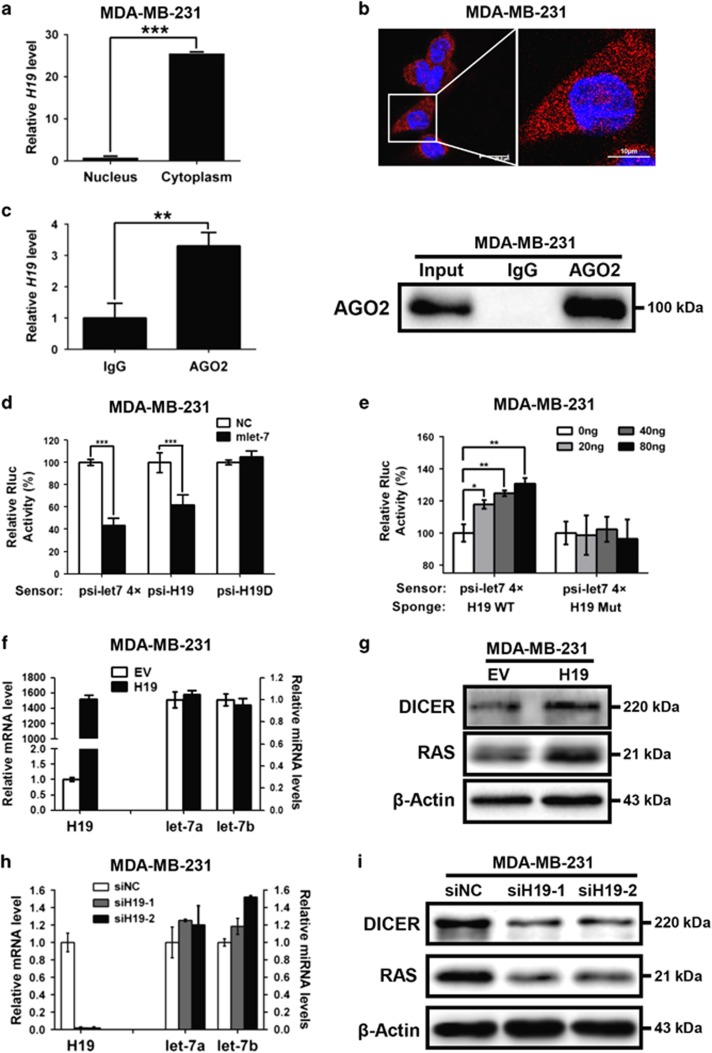
LncRNA-H19 acts as an endogenous miRNA let-7 sponge in breast cancer cells. (**a**) Comparison of the expression of *H19* in cytoplasm and in nucleus by RT-qPCR. Data are represented as mean±S.D. ***P<0.001, *n*=3. (**b**) Shown was the representative image of the *in situ* location of *H19* transcripts in MDA-MB-231 cells. The scale bar represents 20 *μ*m (left) and 10 *μ*m (right). (**c**) Immunoprecipitation using anti-AGO2 antibody (lane 3) or IgG (lane 2) followed by western blot analysis using a mouse monoclonal anti-AGO2 (right). Co-IP with rabbit anti-AGO2 antibody or preimmune IgG from extracts of MDA-MB-231 cells. *H19* RNA levels in immunoprecipitates were determined by RT-qPCR (left). Data were shown as mean±S.D. from three independent experiments, **P<0.01. (**d**) The target validation using luciferase reporters; the indicated constructs were each transfected into MDA-MB-231 cells together with negative control miRNA (NC) or let-7 mimics (mlet-7) at a final concentration of 48 nM. Numbers are mean±S.D. (*n*=3, ***P<0.001). (**e**) Let-7 sensor (psiCHECK2-let-7 4x) was transfected into MDA-MB-231 cells, together with 0, 20, 40 or 80 ng of sponge plasmid wide type *H19* (WT) or mutant *H19* (Mut). Numbers are mean±S.D. (*n*=3, *P<0.05, **P<0.01). (**f**) Empty vector (EV) or full-length *H19* (WT) was transfected into MDA-MB-231 cells. The relative *H19* mRNA level was normalized to ACTB and let-7a/7b miRNA levels were normalized against those of U6B. Numbers are mean±S.D. (*n*=3). (**g**) The protein levels of let-7 targets DICER and RAS were confirmed by western blot. (**h**) MDA-MB-231 cells were transfected with siRNA targeting *H19* (siH19) or negative control RNA (siNC), *H19* expression levels and let-7a/7b miRNA levels were evaluated by RT-qPCR, and (**i**) the protein levels of let-7 targets were detected by western blot

To explore the molecular mechanism whereby H19 regulates BCSCs, we first examined the expression level and location of H19 in breast cancer cells. As shown in Figure 4a (MDA-MB-231 cells) and Supplementary Figure 5A (SK-BR-3 cells), *H19* expressed at higher levels in the cytoplasm than in the nucleus (*P*<0.001). This result was further confirmed by FISH assay, which showed that *H19* transcripts were abundant in the cytoplasm of MDA-MB-231 (Figure 4b) and SK-BR-3 cells (Supplementary Figure 5B). These findings suggested that H19 interacts with miRNAs in the cytoplasm and functions as endogenous sponges for miRNAs. To test this conjecture, we next performed RNA immunoprecipitation (RIP) analysis with antibodies against AGO2 using extracts from MDA-MB-231 cells. The results showed that H19 was elevated in AGO2-containing miRNPs compared with control IgG immunoprecipitates (Figure 4c, left, *P*<0.01). Western blotting confirmed that AGO2 was expressed in the input and the pulled down complexes (Figure 4c, right). These data showed that H19 is recruited to AGO2-related RNA-induced silencing complexes and functionally interacted with miRNAs in breast cancer cells.

Furthermore, let-7 mimics (mlet-7) substantially repressed the luciferase activities of the psiCHECK2-let-7 4 × and psiCHECK2-*H19* (*P*<0.001) reporters, which harbors four and two copies of let-7-binding sites, respectively. By contrary, psiCHECK2-*H19D*, with let-7-binding sites deletion, no longer responded to mlet-7 (Figure 4d). We further generated *H19*-overexpressing plasmids with either wild-type (WT) or mutated (Mut) let-7-binding sites. The psiCHECK2-let-7 4 × reporter was co-transfected with increasing amounts of WT *H19* or Mut *H19* (sponge) into MDA-MB-231 cells. The results showed that the relative let-7 reporter luciferase activity was increased in response to WT *H19* in a dose-dependent manner but remained unchanged with the Mut *H19* plasmids (Figure 4e). Notably, although the expression levels of let-7a and let-7b were unaltered when *H19* was overexpressed in MDA-MB-231 cells (Figure 4f), the protein levels of DICER and RAS targeted by let-7 were increased (Figure 4g). Similarly, let-7a and let-7b displayed no significant changes in expression levels when *H19* was silenced by small interfering RNAs (siRNAs; Figure 4h), whereas the levels of DICER and RAS were markedly decreased in *H19* knockdown cells (Figure 4i). Consistently, similar results were also observed for SK-BR-3 cells upon *H19* overexpression and depletion (Supplementary Figures 5C and D). Taken together, these findings evidently suggest that H19 acts as a miRNA sponge to restrict the biological function of let-7 in breast cancer cells.

### H19 elevates LIN28 expression through a let-7-dependent mechanism

**Figure 5:**
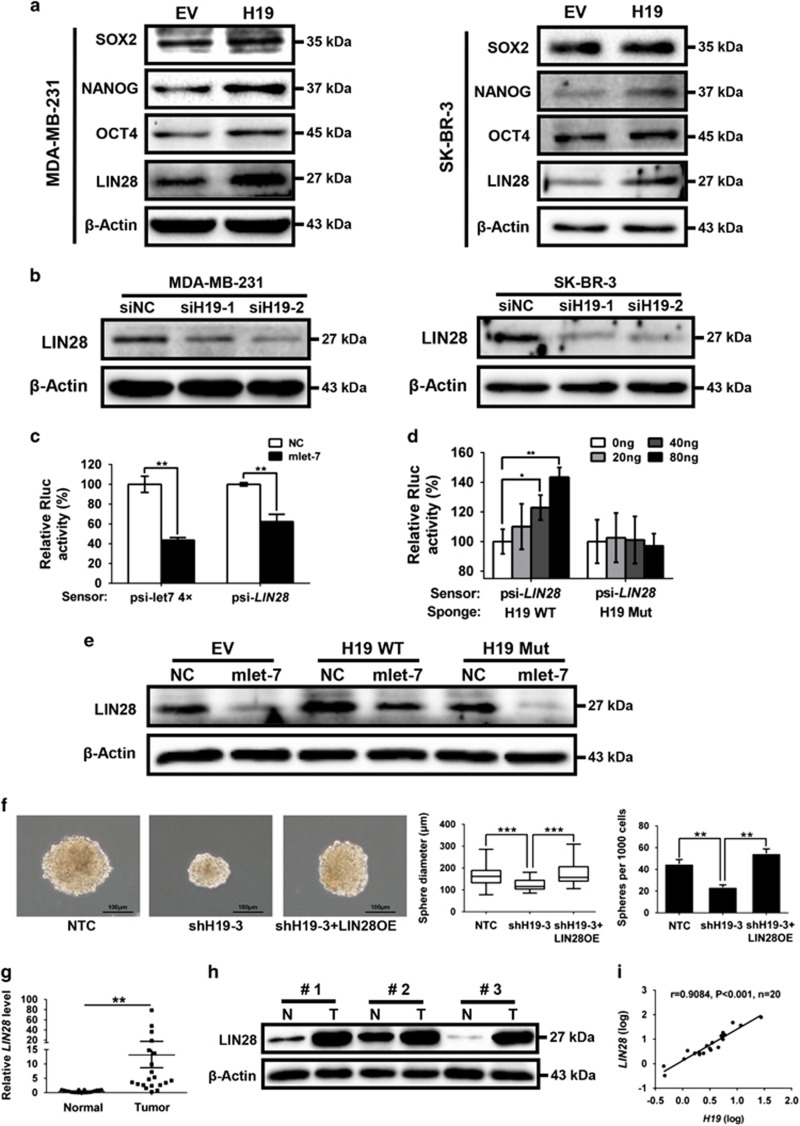
H19/let-7 axis modulates LIN28 expression and CSCs maintenance. (**a**) The protein levels of a panel of core pluripotency factors were evaluated in *H19* overexpression MDA-MB-231 (left) or SK-BR-3 (right) cells. (**b**) The protein level of LIN28 were confirmed by western bolt in *H19* depletion cancer cells. (**c**) H19 shared regulatory let-7 miRNA with LIN28. The target validation was confirmed by using a luciferase reporter (psi-*LIN28*) transfected into MDA-MB-231 cells. Numbers are mean±S.D. (*n*=3, **P<0.01). (**d**) Psi-*LIN28* was transfected into MDA-MB-231 cells, together with 0, 20, 40 and 80 ng of sponge plasmid WT *H19* or Mut *H19*. Numbers are mean±S.D. (*n*=3, *P<0.05 and **P<0.01). (**e**) The protein levels of LIN28 were determined in the indicated groups 48 h post transfection. (**f**) Overexpression of *LIN2*8 rescued lentivirus shH19-mediated reduction of self-renewal in mammosphere formation assays. Numbers are mean±S.D. (*n*=3, **P<0.01 and ***P<0.001). (**g**) Analysis of *LIN28* expression in breast cancerous tissue and adjacent normal tissues (*n*=20). The relative *LIN28* mRNA level was normalized to *ACTB*. The statistical differences were analyzed using the paired *t*-test. (**h**) The protein level of LIN28 in three pairs of randomly chosen clinical specimens was confirmed by western blot. (**i**) Spearman correlation showed positive correlations between expression of *H19* and *LIN28* in a statistically significant manner (*n*=20)

To identify the potential downstream targets of H19 and let-7 involved in BCSC maintenance, we first examined the expression levels of a panel of core pluripotency factors upon *H19* overexpression in breast cancer cells. Interestingly, overexpression of *H19* resulted in a substantial increase of LIN28 protein level in MDA-MB-231 and SK-BR-3 cells (Figure 5a). As *LIN28* was a validated target of let-7, we examined whether LIN28 was regulated by the H19/let-7 axis in breast cancer cells. LIN28 expression was remarkably reduced following *H19* depletion by siRNAs in two breast cancer cells (Figure 5b). In addition, both the levels of LIN28 mRNA (Supplementary Figure 6A) and protein (Supplementary Figure 6B) decreased in the presence of mlet-7 and increased in the presence of let-7 inhibitors (ilet-7). Furthermore, the 3′UTR sequence of *LIN28* was fused to the luciferase-coding region (psiCHECK2-*LIN28*, Supplementary Figure 6C) and transfected into MDA-MB-231 cells together with mlet-7 in parallel with the negative control (NC). The results showed that mlet-7 significantly repressed the relative luciferase activity of reporter psiCHECK2-*LIN28* (Figure 5c, *P*<0.01). However, when we co-transfected psiCHECK2-*LIN28* (sensor) with increasing amounts of WT *H19* (sponge) into MDA-MB-231 cells, the relative luciferase activity was promoted in response to WT *H19*, but Mut *H19*, in a dose-dependent manner (Figure 5d). The effect of H19/let-7 axis in the regulation of LIN28 expression was further investigated by rescue assay shown in Figure 5e. The results revealed that the expression of LIN28 decreased in the presence of mlet-7, whereas it increased in the presence of WT *H19*. Notably, WT *H19* was able to restore synthesis of LIN28 protein, even in the presence of mlet-7. On the contrary, these effects were blocked when Mut *H19* were used. To test whether BCSC maintenance by H19 is dependent on LIN28, *LIN28*stably overexpressing cells were established from H19 knockdown MDA-MB-231 (shH19) cells (Supplementary Figure 6D). *H19* knockdown resulted in a decrease in sphere formation capacity in MDA-MB-231 cells, whereas co-overexpression of LIN28 reversed this reduction (Figure 5f). Similar rescues were found for colony formation and transwell migration when *LIN28* was overexpressed (Supplementary Figures 6E and F). Indeed, protein level of LIN28 was higher in MDA-MB-231 cells compared with the non-cancerous MCF-10A cells (Supplementary Figure 6G). Consistently, *LIN28* expression was also increased in ALDH1^+^ cells and SP cells (Supplementary Figure 6H, *P*<0.001). As shown in Figures 5g and h, the mRNA and protein levels of *LIN28* were significantly higher in breast cancer tissues than corresponding adjacent non-cancerous tissues (*P*<0.01). In addition, *H19* and *LIN28* also displayed a strong correlation in breast patient tumors (Figure 5i, *P*<0.001). Together, these results suggest that H19 acts as a ceRNA to inhibit let-7 function, leading to de-repression of LIN28 that is crucial for the maintenance of BCSCs.

### LIN28 promotes H19 expression by suppressing let-7 production

**Figure 6:**
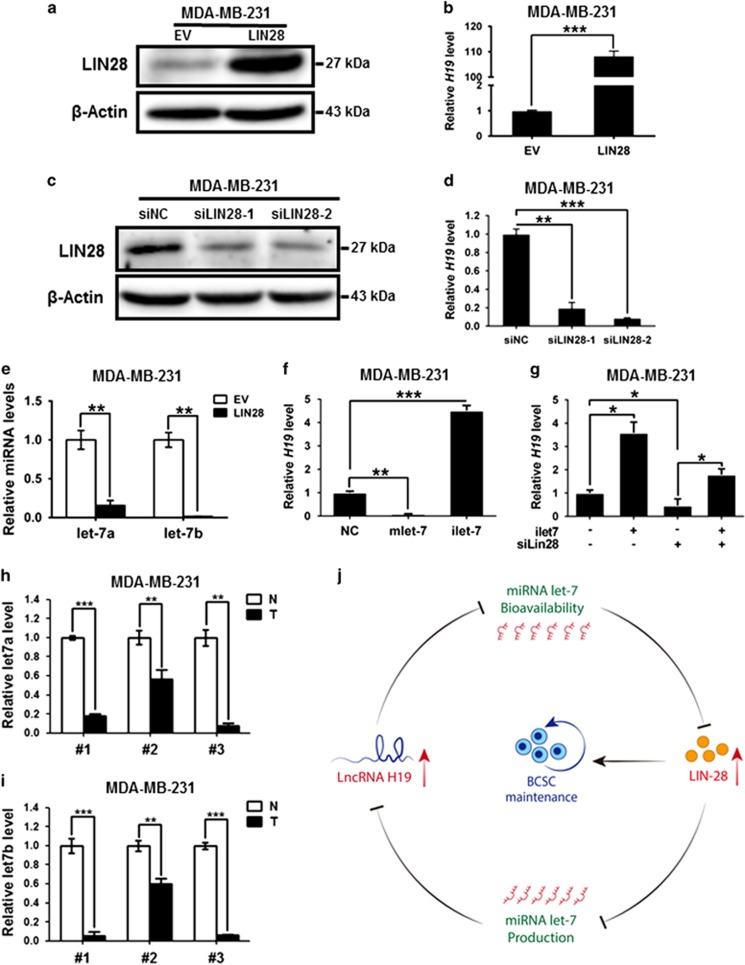
LncRNA-H19 formed a double-negative circuitry with miR-let-7 and LIN28 in breast cancer cells. (**a**) MDA-MB-231 cells were transfected with *LIN28*-overexpressing vector (LIN28) or empty vector (EV), the protein level of LIN28 was quantified by western blot 72 h post transfection. (**b**) The relative *H19* mRNA level was analyzed by RT-qPCR when overexpressed *LIN28*. Numbers are mean±S.D. (*n*=3, ***P<0.001). (**c**) The protein level of LIN28 was detected 72 h after transfection of siRNAs targeting *LIN28* (si*LIN28*) or siNC. (**d**) *LIN28* depletion decreased expression of *H19*. Numbers are mean±S.D. (*n*=3, **P<0.01 and ***P<0.001). (**e**) LIN28-overexpressing vector (LIN28) or empty vector (EV) was transfected into MDA-MB-231 cells. After transfection 48 hours, let7a/7b miRNA levels were measured by RT-qPCR. Numbers are mean ± SD (*n* = 3, **P<0.01). (**f**) MDA-MB-231 cells were transfected with 48 nM control miRNA (NC), let-7 mimics (mlet-7) or let-7 inhibitors (ilet-7). RNAs were extracted 48 h later and RT-qPCR analysis performed. Numbers are mean±S.D. (*n*=3, **P<0.01 and ***P<0.001). (**g**) MDA-MB-231 cells were transfected with the indicated mixture, and the relative *H19* mRNA level was detected by RT-qPCR. Numbers are mean±S.D. (*n*=3, *P<0.05). (**h**and **i**) Let-7a (**h**) or let-7b (**i**) miRNA levels in clinical specimens were assessed by RT-qPCR. Numbers are mean±S.D. (*n*=3, **P<0.01 and ***P<0.001). (**j**) Model for the H19/let7/LIN28 regulatory loop in the modulation of BCSCs maintenance

The co-overexpression of *H19* and *LIN28* led us to ask whether *H19* is also upregulated by LIN28. Ectopic overexpression (Figure 6a) and transient knockdown (Figure 6c) of *LIN28* remarkably increased (Figure 6b) and reduced (Figure 6d) *H19* expression, respectively. Similar results were observed in SK-BR-3 cells (Supplementary Figures 7A–D). These results indicated a reciprocal positive regulation between LIN28 and *H19*. In agreement with the fact that LIN28 is a post-transcriptional repressor of let-7, the let-7a and let-7b miRNA levels were markedly decreased by overexpression of *LIN28* (Figure 6e; Supplementary Figure 7E). Furthermore, mlet-7 significantly repressed the expression of *H19*, ilet-7 remarkably enhanced *H19* expression in both MDA-MB-231 (Figure 6f) and SK-BR-3 cells (Supplementary Figure 7F). These data suggest that H19 is reciprocally inhibited by its target let-7, and that this negative feedback loop can be disrupted by LIN28 through its ability to repress let-7 expression.

Next, we analyzed *H19* expression following *LIN28* knockdown in combination with ilet-7 overexpression. As shown in Figure 6g, ilet-7 increased *H19* expression (*P*<0.05) and *LIN28* knockdown caused a downregulation of *H19* (*P*<0.05), whereas co-transfection of ilet-7 relieved this *H19* downregulation caused by *LIN28* knockdown (*P*<0.05). Similar results were identified in SK-BR-3 cells (Supplementary Figure 7G). As let-7 is suppressed by both H19 and LIN28 in breast cancer cells, we analyzed let-7 expression in three paired breast cancer patient samples. Let-7a and let-7b were substantially downregulated in breast cancer patient tissues compared with the corresponding adjacent non-cancerous tissues (Figures 6h and i). As cancer stem cells tend to cause metastasis in breast tumor, we further examined the expression of H19, LIN28 and let-7 in metastatic and non-metastic mammary tumors from twenty tumor tissues stratified on clinical progression. Intriguingly, the results showed that H19 and LIN28 levels were highly expressed in metastasis tumors, whereas let-7a and let-7b levels were much lower in metastasis tumors than non-metastasis tumors (Supplementary Figures 7H-J). In conclusion, our study establishes that H19/let-7/LIN28 form a double-negative feedback circuitry to regulate BCSC maintenance (Figure 6j).

## Discussion

In present study, we demonstrate that H19-let-7-LIN28 exhibit a double-negative feedback loop in regulation of BCSC maintenance. In agreement, *H19* is significantly elevated in breast tumors and BCSC-enriched populations (Figure 1). Moreover, H19 has a critical role in promoting BCSC properties and tumorigenesis *in vitro* and *in vivo* (Figures 2 and 3). Here we describe a mechanism in which H19 functions as a ceRNA to sponge let-7 family of microRNAs and promotes the expression of core pluripotency factor LIN28 (Figures 4 and 5). Intriguingly, H19 is also repressed by let-7 in a negative-feedback mechanism. In consequence, LIN28 can also indirectly promote *H19* expression through suppressing the miRNA level of let-7 (Figure 6).

Emerging evidence suggests that CSCs exist in many cancers and are closely associated with cancer progression, metastasis and chemoresistance.^10, 32, 33^ To fully explore their potentials as cancer markers and drug targets, a better understanding of the underlying molecular basis of cancer stem cell maintenance is required. As the core pluripotency factors OCT4, SOX2 and c-Myc are regulated by lincRNAs feedback loops,^34, 35^ it is therefore suggested that lincRNA may be involved in maintaining cancer stem cell phenotypes. Consistent with this idea, suppression of *H19* with siRNA in prostate epithelial cells (RWPE-1) decreases colony-forming potential. Conversely, overexpression of *H19* significantly increases sphere-forming capacity.^36^ H19 also promotes soft-agar colony formation in breast cancer cells.^37^ Consistently, our studies demonstrated that BCSC-enriched populations (i.e., ALDH1^+^ subpopulation, mammoshperes, 3D culture cells and SP cells) and breast tumors displayed high lncRNA-*H19* expression. Gain or loss of function analysis further confirmed that H19 is critical for BCSC properties *in vitro*. In addition, previous study also showed that overexpression of *H19* promotes breast tumor progression after subcutaneous injection of *H19*-recombined cells into SCID mice.^37^ Indeed, our findings demonstrated that H19 not only facilitates tumor growth but also elevates tumor-initiating ability in xenograft nude mice model. However, we also found that neither overexpression nor depletion of *H19* had effect on cell proliferation in breast cancer cells, indicating that spheroid formation, anchorage-independent colony formation and tumor-initiating abilities regulated by H19 are linked to self-renewal and not proliferation.

Subcellular localization is often a useful predictor for the function and mechanism of action of lincRNAs. For example, nuclear lincRNAs need to be associated with chromatin-remodeling complexes to regulate transcription, e.g., HOTAIR.^38^ In addition, cytoplasmic lincRNAs mostly function as endogenous ‘sponges' for miRNAs and act as post-transcriptional regulators,^39^ such as LincROR.^40^ Recent findings revealed that cytoplasmic H19 functions as an endogenous sponge for the let-7 family of microRNAs to regulate cancer metastasis^41^ and muscle metabolism.^42^ In our study, H19 localized in the cytoplasm is associated with AGO2 and acts to sponge let-7 to inhibit its bioactivity in breast cancer cells. Furthermore, H19 functions as a novel upstream regulator of the core pluripotency factor LIN28 and protects LIN28 from let-7-mediated degradation. Intriguingly, LIN28 also suppresses the production of let-7. H19 is downregulated by its target miRNA let-7 in non-diabetic muscle,^42^ myotubes^43^ and breast cancer cells. In consequence, an accumulation of LIN28 can indirectly elevate H19 expression through inhibiting let-7. Thus, we identify a novel negative-feedback mechanism between H19 and let-7, as well as a double-positive feedback loop between H19 and LIN28 mediated through let-7 in breast cancer.

In general, feedback loops are widely observed between miRNAs and their targets. They allow the regulatory network to maintain bi-stable states.^44^ In a previous study, miR-489 and HER2-SHP2-MAPK signaling axis form a double-negative feedback loop that can regulate breast cancer cell proliferation and tumor progression.^45^ A double-negative feedback loop involving ZEB2 and miR-145 has a critical role in EMT and stemness maintenance during bone metastasis of prostate cancer cells.^46^ Here we reveal that lncRNA-H19, functioning as a ceRNA, links the network of miRNA let-7 and core transcription factor LIN28. H19 overexpression in breast cancer breaks the homeostatic balance between let-7 and LIN28, leading to the inhibition of let-7 and subsequent elevation of LIN28. Furthermore, accumulation of LIN28 not only activates downstream signaling to promote self-renewal, but also reciprocally increases the H19 expression. This novel regulatory mechanism establishes a comprehensive regulatory network that adapts to environmental changes in the maintenance of BCSCs. Importantly, both quality and quantity of let-7 are dual-suppressed in double-negative feedback loop. In addition, let-7 displays a lower expression in breast tumor tissues compared with adjacent tissues. As let-7 miRNA is a key tumor suppressor that targets numerous oncogenes such as c-Myc and RAS,^47, 48^ this double ‘beat' for let-7 releases its oncogenic factors and underlines BCSC maintenance.

In summary, our studies reveal that lncRNA–miRNA–mRNA double-negative feedback loop constitutes a new dimension of post-transcriptional gene regulation in BCSCs. H19 functions as a ceRNA to constitute a dual-feedback regulatory circuitry with let-7 and LIN28. This bi-directional regulation network reflects a novel mechanism with a vital role in BCSC maintenance. Targeting this newly identified regulatory circuitry provides therapeutic opportunities for aggressive breast cancers.

## Materials and methods

### Clinical samples, cell lines and primary breast cancer cell isolation

All breast cancer samples were obtained from newly diagnosed patients with prior patients consent and the approval of the Institutional Clinical Ethics Review Board of the first Affiliated Hospital of Dalian Medical University. Samples were frozen in liquid nitrogen for mRNA and protein extraction. Overall survival was defined as the period from the date of diagnosis to the date of death. Human breast cancer cell lines MDA-MB-231, SK-BR-3 and MCF-7 cells were maintained in Dulbecco's modified Eagle's medium (DMEM, Invitrogen, Carlsbad, CA, USA) supplemented with 10% fetal bovine serum (Gibco, Carlsbad, CA, USA), respectively. BT-549 cells were maintained in RPMI-1640 (Invitrogen) supplemented with 10% fetal bovine serum. MCF-10A cells were cultured in DMEM/F12 medium (Invitrogen) supplemented with 5% horse serum (HyClone, Logan, UT, USA), 20 ng/ml EGF (Sigma-Aldrich, St Louis, MO, USA), 100 ng/ml cholera toxin (Sigma-Aldrich), 100 ng/ml insulin (Sigma-Aldrich) and 500 ng/ml hydrocortisone (Sigma-Aldrich). HEK293T cells were routinely cultured in DMEM medium supplemented with 10% fetal bovine serum. All cells were incubated at 37 °C in a humidified incubator containing 5% CO_2_. All cell lines were obtained from the American Type Culture Collection (ATCC, Manassas, VA, USA). The cell lines were authenticated at American Type Culture Collection before purchase by their standard short tandem repeat DNA typing methodology. For primary breast cancer cell isolation, the tumor xenografts were mechanically and enzymatically dissociated to yield clumps of epithelial cells by incubation at 37 °C for 2 h in a 1:1 solution of collagenase I (3 mg/ml): hyaluronidase (100 U/ml; Sigma-Aldrich). After filtration through a 40 *μ*m pore filter and washing with PBS, the tumor tissues were trypsin dissociated to single cells for subsequent experiments.

### Fluorescence-activated cell sorting

For ALDH1 assay, the ALDH1^+^ population was detected by ALDEFLUOR kit (Shanghai Stem Cell Technology Co. Ltd, Shanghai, China) following manufacturer instructions. MDA-MB-231 cells (1 × 10^6^ per ml) were analyzed on a BD FACScalibur flow cytometer (BD Biosciences, Franklin Lakes, NJ, USA) after staining in ALDH1 substrate containing assay buffer for 30 min at 37 °C. The negative control was treated with diethylaminobenzaldehyde, a specifc ALDH1 inhibitor. ALDH1^**+**^ or ALDH1^**−**^ cells (at least 1 × 10^6^) were collected for RNA extraction. For SP cells sorting, MDA-MB-231 cells (1 × 10^6^ per ml) were incubated with 2 *μ*m FTC, an ABCG2-specifc inhibitor, for 20 min in negative control tubes before adding Hoechst 33342 (Sigma-Aldrich). Then, MDA-MB-231 cells were incubated in Hanks' balanced salt solutions supplemented with 2% FBS, 10 mM HEPES, 5 *μ*g/ml Hoechst 33342 for 90 min at 37 °C with mixing every 15 min, followed by washing with cold growth medium. Cells were resuspended at a concentration of 2 × 10^6^ per ml. Then, PI was added to a final concentration of 2 *μ*g/ml to discriminate dead cells from live cells. The gating of side population was based on negative controls in which FTC was used. Finally, cells were analyzed on a BD FACScalibur flow cytometer and collected at least 1 × 10^6^ SP cells or non-SP cells for RNA extraction.

### Fluorescent *in situ* hybridization

A fragment of *H19* designed as its probe was used and labeled with digoxigenin (DIG)-UTP (Roche, Mannheim, Germany) using the mMESSAGE T7 Ultra *In Vitro* Transcription kit (Ambion, Austin, TX, USA) in accordance with the manufacturer's directions. Slides were hybridized with probes overnight, washed twice with 50% formamide/2 × saline sodium citrate (SSC) and twice with 2 × SSC at 50 °C for 5 min each, then incubated with 1:500 diluted sheep anti-Dig (lnvitrogen) for 1 h at 37 °C, followed by counterstained with DAPI (1 *μ*g/ml), visualized using a confocal microscope (Leica, Wetzlar, Germany). Probe sequences were listed in Supplementary Table 1.

### Plasmids construction and stable cell lines generation

Flag-LIN28 was built as described by Qiu *et al.*^49^ Wild-type human *H19* (WT *H19*) and mutant human *H19* (Mut *H19*) were constructed as previously described.^43^ psiCHECK2-*H19* and psiCHECK2-*H19D* with or without two copies of predicted that let-7-binding sites were established as described by Kallen *et al*,^43^ psiCHECK2-let7 4 × was built as described by Iwasaki *et al.*^50^ To make psiCHECK2-*LIN28*, the bioinformatics tool Miranda (http://www.microrna.org) was used to search for let-7-binding sites in the full-length transcripts of *LIN28* and predicted let-7-binding sites within *LIN28* 3′-UTR fragments were obtained by RT-PCR and were inserted into the luciferase reporter vector psiCHECK2 (Promega, Madison, WI, USA) between *Xho*1 and *Not*1 sites. *H19* and *LIN28* cloned from WT *H19* and Flag-*LIN28* were inserted in pLVX-DsRed-N1-Monomer (Clontech, Mountain View, CA, USA) between *Bam*H1 and *Not*1 sites to construct pLVX-*H19*-DsRed and pLVX-*LIN28*-DsRed. The pLVX-*H19*-DsRed and pLVX-*LIN28*-DsRed lentivirus were packaged in HEK293T cells and viral particles were collected 48 h post transfection. After infection, the cells stably expressing *H19* and *LIN28* were chosen by selection with 2 *μ*g/ml puromycin (Sigma-Aldrich), respectively. For shRNA lentiviruses (GenePharma, Suzhou, China) infection, cells were infected in six-well plates and subsequently split into 10 cm dishes in the presence of 2 *μ*g/ml puromycin for selection over 72 h. All primer sequences were listed in supplementary table 1.

### siRNAs, microRNA mimics and microRNA inhibitors transfection

Transient transfection was performed by using Lipofectamine 2000 (Invitrogen) according to the manufacturersection, cel. The following reagents were used: siRNAs specifically targeting *H19*, *LIN28* and siRNA control were purchased from GenePharma, and miR-let7a or miR-let7b mimics, Pre-miR negative control, miR-let7a or miR-let7b inhibitors and anti-miR control were purchased from Qiagen, Valencia, CA, USA (let-7a, cat. no. MS00006482, let-7b, cat. no. MS00003122). All primer sequences were listed in Supplementary Table 1.

### Mouse mammary tumor model and xenograft tumor formation

Mice carrying the beta-lactoglobulin Cre (BLG-Cre) transgene, homozygous for floxed exons 22–24 of the breast cancer 1 (Brca1) allele, and heterozygous for p53 tumor suppressor gene (Trp53) deficiency were bred (The Jackson Laboratory, Mouse strain datasheet-012620, Bar Harbor, ME, USA). After two rounds of pregnancy, tumors were allowed to grow and used for subsequent experiments. For xenograft model, MDA-MB-231-shCtrl or MDA-MB-231-shH19 cells (6 × 10^6^) in 100 *μ*l matrigel were subcutaneously injected at the right or left dorsal fank of female nude mice (4–6 weeks), respectively. The body weight of the animals and the two perpendicular diameters (*a* and *b*) were recorded every 3 days. Tumor volume (*V*) was calculated according to the following formula: *V*=(*a* × *b* × *b*)/2. After 6 weeks, tumor mass was resected and dissociated to form tumor suspension for the secondary serial tumor transplantation. The protocol was performed as previous described. Then, 1 × 10^5^, 1 × 10^4^, 1 × 10^3^ and 1 × 10^2^ tumor cells isolated from the first tumor xenografts were injected into nude mice. Subsequent steps were similar with the first tumor transplantation. Investigation was conducted in accordance with the ethical standards and according to the Declaration of Helsinki and national and international guidelines approved by the institutional animal care and use committee of Dalian Medical University.

### Luciferase reporter assay

MDA-MB-231 cells (1 × 10^4^) were seeded into each well of 48-well-plate and the following steps were carried out as previously described.^49^ MDA-MB-231 cells were co-transfected with 10 ng of the indicated luciferase reporter and 48 nM miRNA mimics (Qiagen, Valencia, CA, USA) using Lipofectamine 2000 (Invitrogen). Eighteen hours after transfection, luciferase activity was monitored using the Dual-Luciferase Reporter Assay System (Promega) and a luminometer (Molecular Devices, Sunnyvale, CA, USA). Renilla luciferase activity was normalized against firefly luciferase activities and presented as percentage of inhibition. Results represented the average of triplicate samples from three independent experiments.

### Immunoprecipitation assay

The RIP experiment was carried out as previously described^43^ using rabbit anti-AGO2 antibody (Abcam, Cambridge, MA, USA) on extracts of breast cancer cells MDA-MB-231. The co-precipitated RNAs were extracted with Trizol reagent (Invitrogen) and detected by RT-qPCR. The specificity of the AGO2 antibody was confirmed by immunoprecipitation (IP). MDA-MB-231 cells (1 × 10^7^) were lysed in the soft lysis buffer. Rabbit anti-AGO2 antibody (2 *μ*g)- or control IgG (2 *μ*g)-coated magnetic beads was added to each binding reaction tube and incubated at 4 °C for 4 h. Beads were washed five times with the washing buffer and boiled in the loading buffer for 10 min. The proteins were subjected to SDS-PAGE and detected by mouse anti-AGO2 antibody (Millipore, Billerica, MA, USA).

For 3D culture, sphere cells culture, transwell migration assay, cell plate colony formation assay, sphere formation assay, RNA extraction RT-PCR and real-time PCR analysis, western blot assay, proliferation assay and statistical analysis, see Supplementary Materials and Methods.
