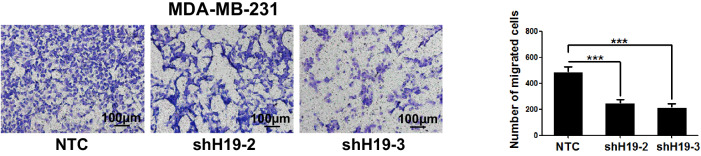# Correction: H19/let-7/LIN28 reciprocal negative regulatory circuit promotes breast cancer stem cell maintenance

**DOI:** 10.1038/s41419-024-06793-5

**Published:** 2024-08-27

**Authors:** Fei Peng, Ting-Ting Li, Kai-Li Wang, Guo-Qing Xiao, Ju-Hong Wang, Hai-Dong Zhao, Zhi-Jie Kang, Wen-Jun Fan, Li-Li Zhu, Mei Li, Bai Cui, Fei-Meng Zheng, Hong-Jiang Wang, Eric W.-F. Lam, Bo Wang, Jie Xu, Quentin Liu

**Affiliations:** 1 Institute of Cancer Stem Cell, Dalian Medical University, Dalian; State Key Laboratory of Oncology in South China, Cancer Center, Sun Yat-sen University, Guangzhou, 510060 China; 2 Department of Breast Surgery, The Second Affiliated Hospital of Dalian Medical University, Dalian, 116023 China; 3 Department of Oncology, The First Affiliated Hospital of Dalian Medical University, Dalian, 116011 China; 4 Department of Hematology, The Second Affiliated Hospital of Dalian Medical University, Dalian, 116023 China; 5 Department of Obstetrics and Gynaecology, The First Affiliated Hospital of Dalian Medical University, Dalian, 116011 China; 6 Department of Medical Oncology, The Eastern Hospital of The First Affiliated Hospital, Sun Yat-Sen University, Guangzhou, 510700 China; 7 Department of Breast Surgery, The First Affiliated Hospital, Dalian Medical University, Dalian, 116011 China; 8 Department of Surgery and Cancer, Imperial College London, London, W12 0NN UK

Correction to: *Cell Death and Disease* 10.1038/cddis.2016.438, published online 19 January 2017

The originally published version of this article contains an image misplacement in which the representative image of shH19-3 group was shown (Figure 2g, left panel). The corrected panel and the revised raw images have been provided. The authors previously used the correct raw images to make statistical analysis, however, shown with the misplaced representative image of shH19-3 group in Figure 2g (left panel). Therefore, the right panel of Figure 2g is correct, and the correction in the left panel of Figure 2g does not affect the interpretation or conclusions of this figure. The original article has been corrected.