# Supplementary material for: H19/let-7/LIN28 reciprocal negative regulatory circuit promotes breast cancer stem cell maintenance
Source: Cell Death Dis. 2017 Jan 19;8(1):e2569–. doi: 10.1038/cddis.2016.438 (PMC5386357; doi:10.1038/cddis.2016.438)
Supplement: Supplementary Information [file cddis2016438x1.docx]

**Supplementary Materials and Methods**

*Three-dimensional culture and sphere cells culture*

Eight-chambered RS glass slides (BD Falcon, USA) were pre-coated with 50μL growth factor reduced MatrigelTM (BD Biosciences, USA) per well. The matrigel was solidiﬁed at 37°C for 30 minutes. Next, the cells were suspended in growth medium containing 2% matrigel and plated at a density of 2×10^3^ cells per well. The cells were fed with growth medium containing 2% matrigel every 4 days. After 10 days of culture, the cells were digested from matrigel with 0.25% EDTA-trypsin. Then collected the cells and performed RNA extraction. For sphere cells culture, dissociated single cells were seeded into ultralow attachment plates with serum-free DMEM/F-12 (Invitrogen, USA) medium supplemented with 2% B27 (lnvitrogen, USA), 20ng/mL bFGF (lnvitrogen, USA), and 20ng/mL EGF (lnvitrogen, USA) and cultured at 37°C in 5% CO_2_. After one month, collected the sphere cells by centrifuge and performed RNA extraction.

*Transwell migration assay*

For migration assay, cells (5×10^4^) in experimental group were resuspended in culture medium with the same concentration of control group and placed into uncoated membrane in the upper chamber (24-well insert, 8μm, Corning Costar, China). Growth medium supplemented with 10% FBS was used as an attractant in the lower chamber. After being incubated for 24 hours, cells migrated through the membrane were fixed with 4% paraformaldehyde (Santa Cruz, USA) and stained with 0.5% crystal violet (Shanghai Sengon Company, China). The stained cell images were captured by microscope (Olympus, Japan), and five random fields at 10× magnification were counted.

*Cell plate colony formation assay*

Single cells were resuspended and plated in 6 cm dishes at 1000 cells/dish. Cells were incubated at 37°C, 5% CO_2_. Eight to eleven days later, the cells were fixed and stained with 1% crystal violet (Shanghai Sengon Company, China). The number of colonies, defined as >50μm cells/colony were counted. Triplicate dishes were set up.

*Sphere formation assay*

Sphere formation was performed in ultralow attachment plates (Corning Costar) with dissociated single cells (1,000) were seeded in 6-well ultra-low attachment plates with serum-free medium supplemented with 2% B27, 20ng/mL bFGF, and 20ng/mL EGF and cultured at 37°C in 5% CO_2_. After 14 days, the spheres greater than 50μm diameters were counted at 40× magnification under Olympus microscope (Olympus, Japan). The number and diameter of spheres were calculated by Image pro plus 6.0.

*RNA extraction, RT-PCR and real-time PCR analysis*

Total RNA was extracted from breast tissue specimens or cells using the Trizol reagent (Invitrogen, USA) following manufacturer’s instructions. The cDNA was generated with an oligo-dT primer by using SuperScript III RT System (Invitrogen, USA). RT-qPCR was performed using Platinum SYBR Green QPCR SuperMix (Invitrogen, USA) as recommended by the manufacturer. β-actin was used as the internal control. For the cytoplasmic and nuclear RNA extraction, the cytoplasmic and nuclear RNA were respectively isolated from cancer cells using the PARIS^TM^ kit Protein and RNA Isolation System (Ambion, USA) according to the manufacturer’s directions, and following steps were similar as previously described. The cytoplasmic mRNA levels were normalized against ACTB and the nuclear mRNA levels were normalized against XIST. For miRNA extraction, total RNAs were extracted from breast tissue specimens or cells using miRNeasy Mini kit (Qiagen, USA). Levels of mature miRNAs were determined by RT-qPCR using miScript reverse transcription kit (Qiagen, USA) and miScript SYBR Green PCR kit (Qiagen, USA) according to the manufacturers’ instructions. PCR primer sets specific for let-7a or let-7b were purchased from Qiagen, and the indicated miRNA levels were normalized against snRNA RNU6B.

*Western blot*

Cellular or tissue samples were lysed with RIPA buffer with freshly added cocktail protease inhibitor (Thermo Fisher Scientific, USA). An equal amount of protein was separated by SDS-PAGE and transferred to a NC membrane (Millipore, USA). The membranes were blocked with 5% fat-free milk in TBST at room temperature for 1 hour and incubated at 4°C overnight with primary antibodies, followed by incubation with appropriate secondary antibodies (Thermo Fisher Scientific, USA) at room temperature for 60 minutes. Antibody binding was detected with an enhanced chemiluminescence kit (Amersham, UK) using Bio- Rad ChemiDoc XRS+ Imaging System according to the manufacturer’s instructions. The Primary antibodies were used as follows: β-ACTIN (Santa Cruz Biotechnology, USA), SOX2 (Cell signaling technology, USA), OCT4 (Cell signaling technology, USA), NANOG (Abcam, USA), DICER (Cell signaling technology, USA), RAS (Cell signaling technology, USA) and LIN28 (Abcam, USA).

*Proliferation assay*

Dissociated cells (2×10^3^) were seeded in triplicate in 96-well plates. At day 2, ten microliters of sterile cell counting kit-8(cck-8) (Beyotime, China) were added to each well and incubated for 3h at 37℃. The optical density values were determined at least in triplicate against a reagent blank at a test wavelength of 450nm and reference wavelength of 630nm.

*Statistical analysis*

Data were expressed as mean ± SD of three independent experiments with GraphPad Prism software. Statistical analysis was performed using Statistical Package for Social Sciences (SPSS) software (version 16.0). The Student’s t-test was used to make a statistical comparison between groups. p<0.05 was considered statistically significant.
