# Supplementary material for: H19/let-7/LIN28 reciprocal negative regulatory circuit promotes breast cancer stem cell maintenance
Source: Cell Death Dis. 2017 Jan 19;8(1):e2569–. doi: 10.1038/cddis.2016.438 (PMC5386357; doi:10.1038/cddis.2016.438)
Supplement: Supplementary Information [file cddis2016438x2.docx]

**Supplementary Figure Legends**

*Supplementary Figure 1 related to Figure 1*

(A) *H19* levels were measured in four breast cancer cell lines by RT-qPCR, and normal human breast epithelial cells (MCF-10A) were used as controls. Error bars represent the mean ± SD of triplicate experiments (up). MCF-10A and MDA-MB-231 cells were subjected to western blotting to analyze the expression of stemness related factors OCT4, SOX2 and NANOG (down). (B) Relative mRNA levels of *POU5F1, SOX2* and *NANOG* in ALDH1^+^ or SP subpopulations were determined by RT-qPCR and mRNA levels were normalized against ACTB. Numbers are mean ± SD (n=3). Relative mRNA levels of *H19* (C) or *POU5F1, SOX2* and *NANOG* (D) in spheroid cells compared to non-spheroid cells were confirmed by RT-qPCR. (E-F) Comparison of *H19* expression in MDA-MB-231 cells in 2D and 3D culture. Numbers are mean ± SD (n=3). Expression levels are represented as fold changes compared to these of non-spheroid cells. Numbers are mean ± SD (n=3).

*Supplementary Figure 2 related to Figure 2*

(A) Ectopic expression of *H19* mRNA expression was confirmed by RT-qPCR after lentivirus infection in SK-BR-3 cells. (B-D) Ectopic overexpression of *H19* enhanced clonogenicity, migration and mammosphere-forming abilities. Representative images were presented, respectively. Data were shown as mean ± SD from 3 independent experiments, ****p*<0.001. (E) The interfering efficiency of the lentivirus encoding *H19*-targeting shRNAs (shH19) in SK-BR-3 cells were confirmed by RT-qPCR, compared to negative control lentivirus (NTC). (F-H) Knockdown of *H19* reduced breast cancer cells clonogenicity, migration and mammosphere-forming abilities. Data were shown as mean ± SD from triple independent experiments, ***p*<0.01 and ****p*<0.001, respectively.

*Supplementary Figure 3 related to Figure 2*

(A-B) Cell proliferation ability was measured by CCK-8 assay in *H19-*overexpression or *H19*-depletion MDA-MB-231 cells. (C-D) Cell proliferation ability was measured by CCK-8 assay in *H19-*overexpression or *H19*-depletion SK-BR-3 cells.

*Supplementary Figure 4 related to Figure 3*

(A)The knockdown efficiency of *H19* in the 1^st^ tumor xenografts were detected by RT-qRCR. Error bars represent mean ± SD of triplicates. (B-D) The cancer cells isolated from the 1^st^ *H19*-knockdown tumor xenografts displayed the reduced clonogenicity, migration and mammosphere-forming abilities. Representative images were presented, respectively. Data were shown as mean ± SD from triple independent experiments, ***p*<0.01 and ****p*<0.001, respectively.

*Supplementary Figure 5 related to Figure 4*

(A) Comparison of *H19* expression in SK-BR-3 cells in cytoplasm and nucleus by using RT-qPCR. Numbers are mean ± SD (n=3), ***p<0.001. (B) Shown was the representative image of the in situ location of *H19* transcripts in SK-BR-3 cells. The scale bar represents 10μm. (C) SK-BR-3 cells were transiently transfected with empty vector (EV) or full-length *H19* vector (WT). The relative *H19* mRNA level was normalized to ACTB and let7-a/7b miRNA levels were normalized against those of U6B. Numbers are mean ± SD (n=3，left). The protein levels of let-7 targets in SK-BR-3 cells were confirmed by western blot (right). (D) SiRNA targeting linc-*H19* (siH19) and siNC was transfected into SK-BR-3 cells, respectively. H19 expression levels and let-7a/7b miRNA levels were measured by RT-qPCR (left), and protein levels of let-7 targets were detected by western blot (right). Representative gel images were presented. Numbers are mean ± SD (n=3).

*Supplementary Figure 6 related to Figure 5*

MDA-MB-231 cells were transfected with 48nM control miRNA (NC), mlet-7 or ilet-7. The relative *LIN28* mRNA levels were analyzed by RT-qPCR (A) and the protein levels of LIN28 were confirmed by western blot (B). Numbers are mean ± SD (n=3, **p*<0.05 and ****p*<0.001). (C) The constructs of psiCHECK2-*LIN28*. (D) The relative mRNA levels of *LIN28* in the indicated stable cell lines were measures by RT-qPCR. (E-F) Overexpression of *LIN28* rescued LV-shH19 mediated reduction of clonogenic growth and migration ability in colony formation and transwell migration assays. Numbers are mean ± SD (n=3, **p*<0.05 and ***p*<0.01). (G) The protein levels of LIN28 were evaluated in normal breast epithelial cell line MCF-10A and breast cancer cell line MDA-MB-231. (H) Comparison of *LIN28* expression in ALDH1 positive (ALDH1^+^) versus ALDH1 negative (ALDH1^-^) subpopulations (black columns) or SP versus non-SP cells (white columns). Data are represented as mean ± SD. ****p*<0.001, n = 3.

*Supplementary Figure 7 related to Figure 6*

(A-B) SK-BR-3 cells were transfected with *LIN28*-overexpressing vector (LIN28) or empty vector (EV) for 72 hours. The protein level of LIN28 was quantified by immunoblotting and the relative *H19* mRNA level was analyzed by RT-qPCR. Numbers are mean ± SD (n=3, ****p*<0.01). (C-D) SK-BR-3 cells were transfected with siLIN28 or siNC for 72 hours. Immunoblotting and RT-qPCR were performed to detect the protein level of LIN28 and the relative *H19* mRNA level, respectively. Numbers are mean ± SD (n=3, ***p*<0.01). (E) *LIN28*-overexpressing vector (LIN28) or empty vector (EV) was transfected into MDA-MB-231 cells and SK-BR-3 cells, respectively. After transfection 48 hours, let7a/7b miRNA levels were measured by RT-qPCR. Numbers are mean ± SD (n=3, ***p*<0.01 and ****p*<0.001). (F) SK-BR-3 cells were transfected with 48nM control NC, mlet-7 or ilet-7. RNAs were extracted 48 hours later and RT-qPCR analysis was performed. Numbers are mean ± SD (n=3, **p*<0.05 and ****p*<0.001). (G) SK-BR-3 cells were transfected with the indicated mixture and the relative H19 mRNA levels were analyzed by RT-qPCR. Numbers are mean ± SD (n=3, ***p*<0.01 and ****p*<0.001). (H-I) Breast patient tumors were divided into metastasis (n=11) and non-metastasis (n=9). Let-7, H19 and LIN28 levels were evaluated by RT-qPCR (*p<0.05 and ***p<0.001).

*Supplementary Table 1*

All the sequences of primers, probe, shRNAs, siRNAs, microRNA mimics and microRNA inhibitors were listed.
