# Supplementary figures and images for: H19/let-7/LIN28 reciprocal negative regulatory circuit promotes breast cancer stem cell maintenance
Source: Cell Death Dis. 2017 Jan 19;8(1):e2569–. doi: 10.1038/cddis.2016.438 (PMC5386357; doi:10.1038/cddis.2016.438)

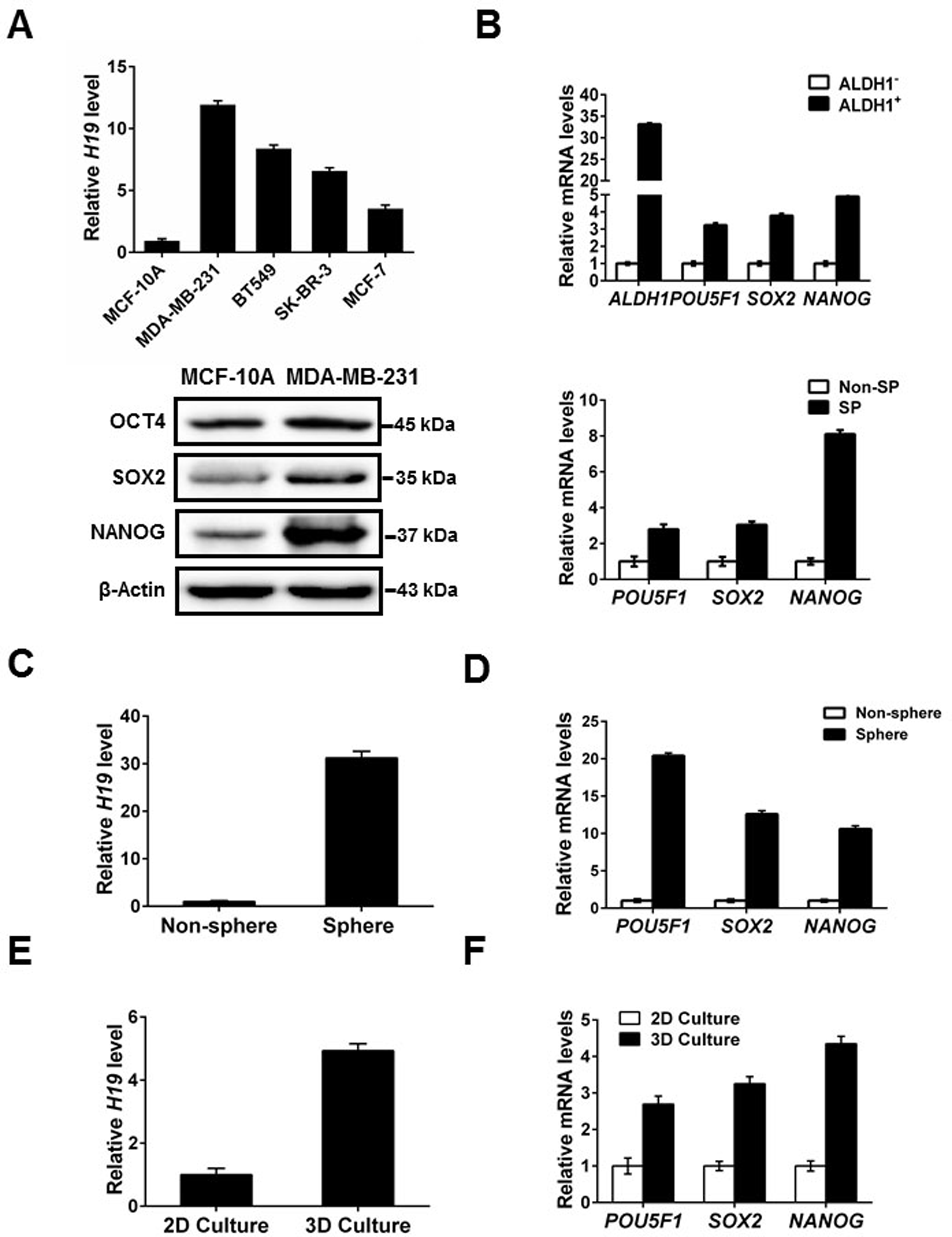

Supplement: Supplementary Table 1 [file cddis2016438x3.tif]

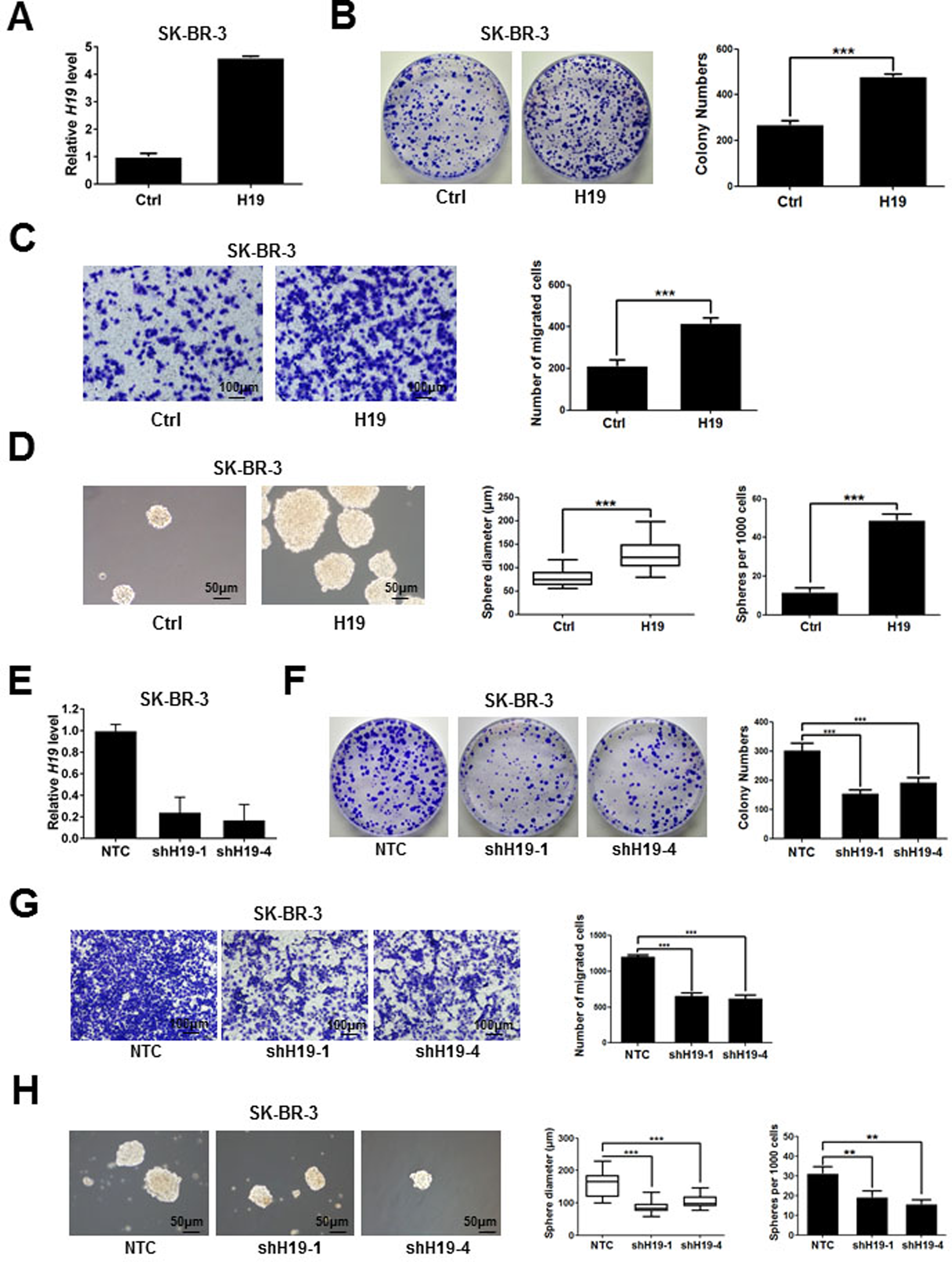

Supplement: Supplementary Figure 1 [file cddis2016438x4.tif]

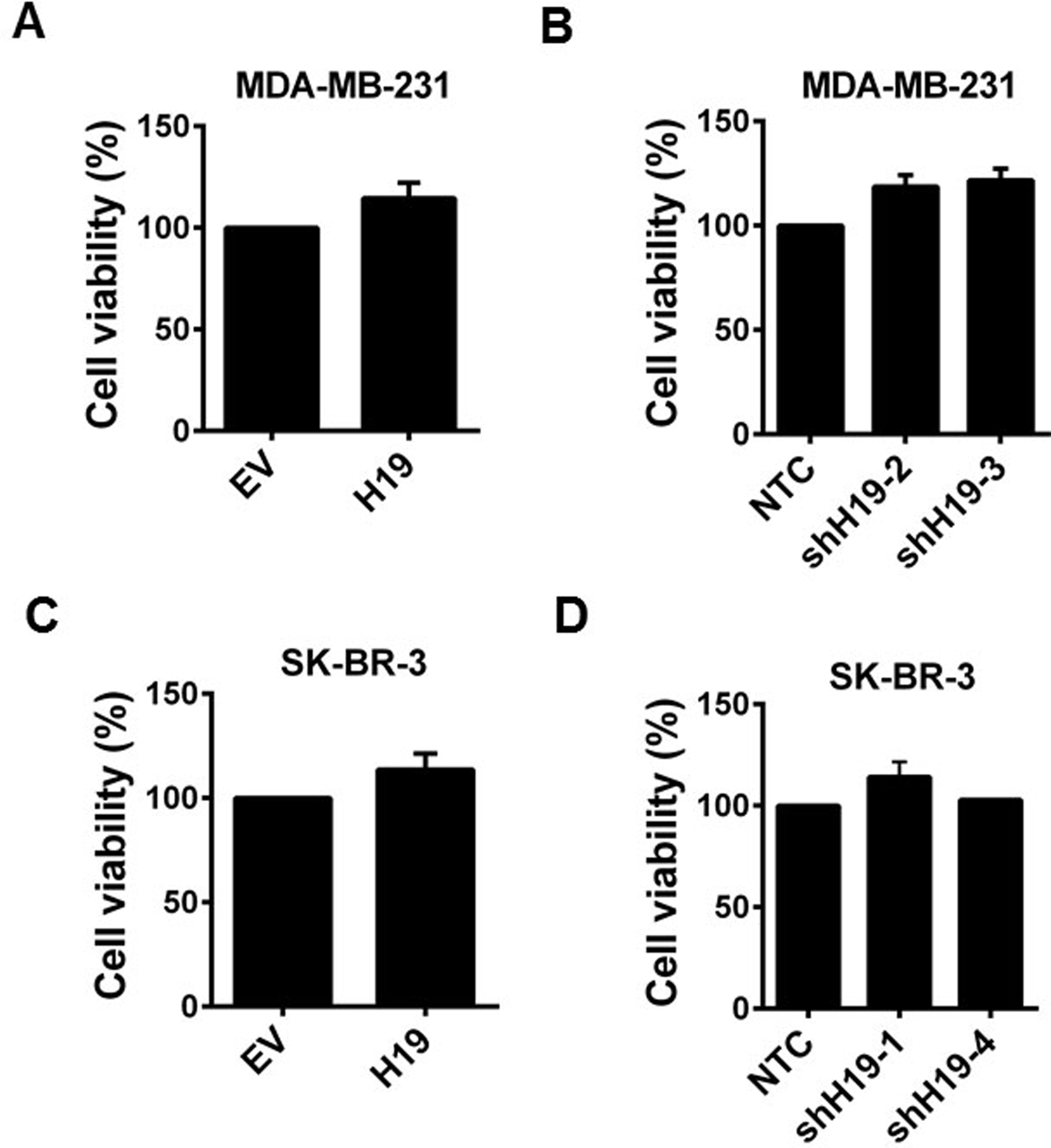

Supplement: Supplementary Figure 2 [file cddis2016438x5.tif]

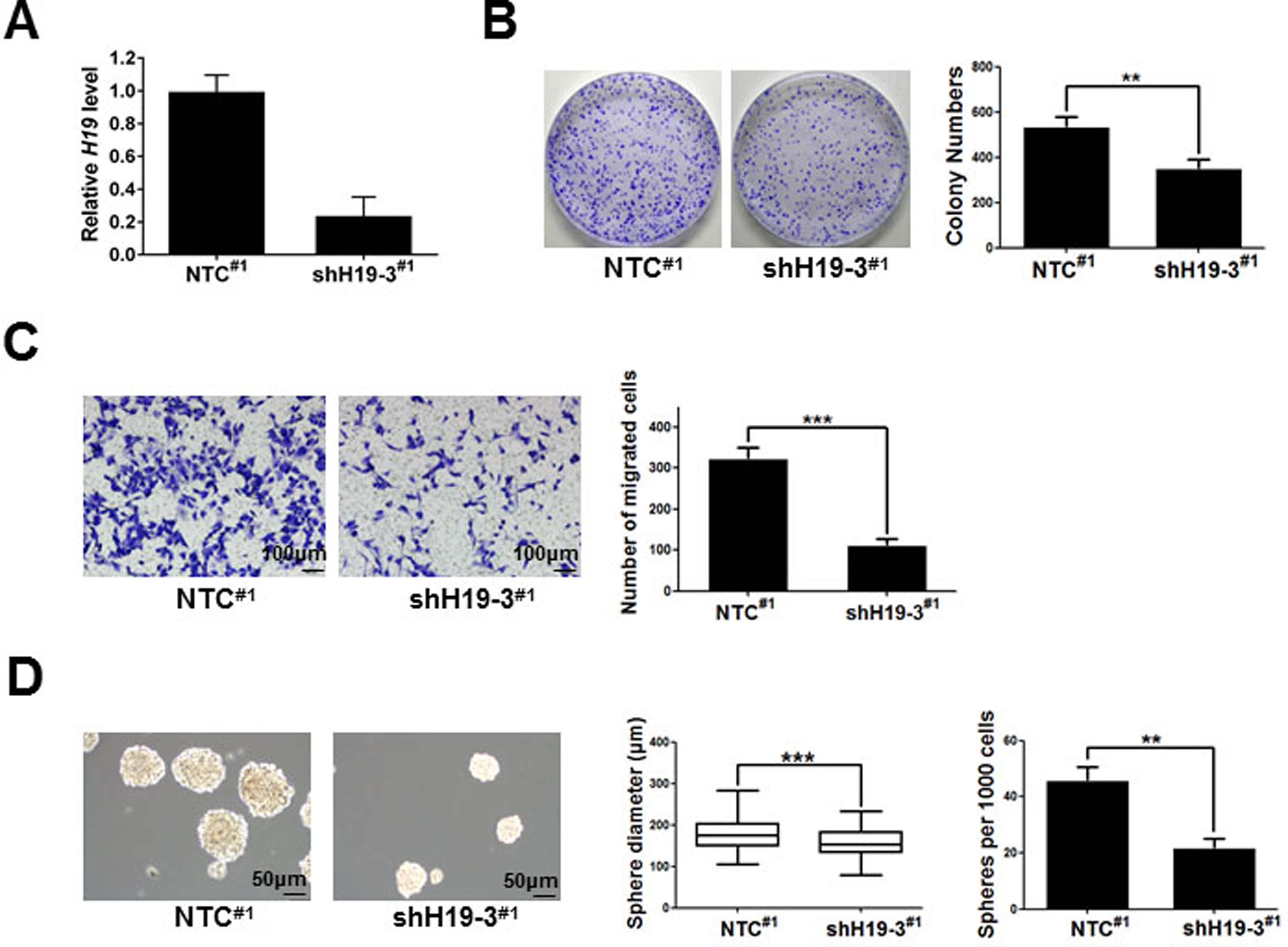

Supplement: Supplementary Figure 3 [file cddis2016438x6.tif]

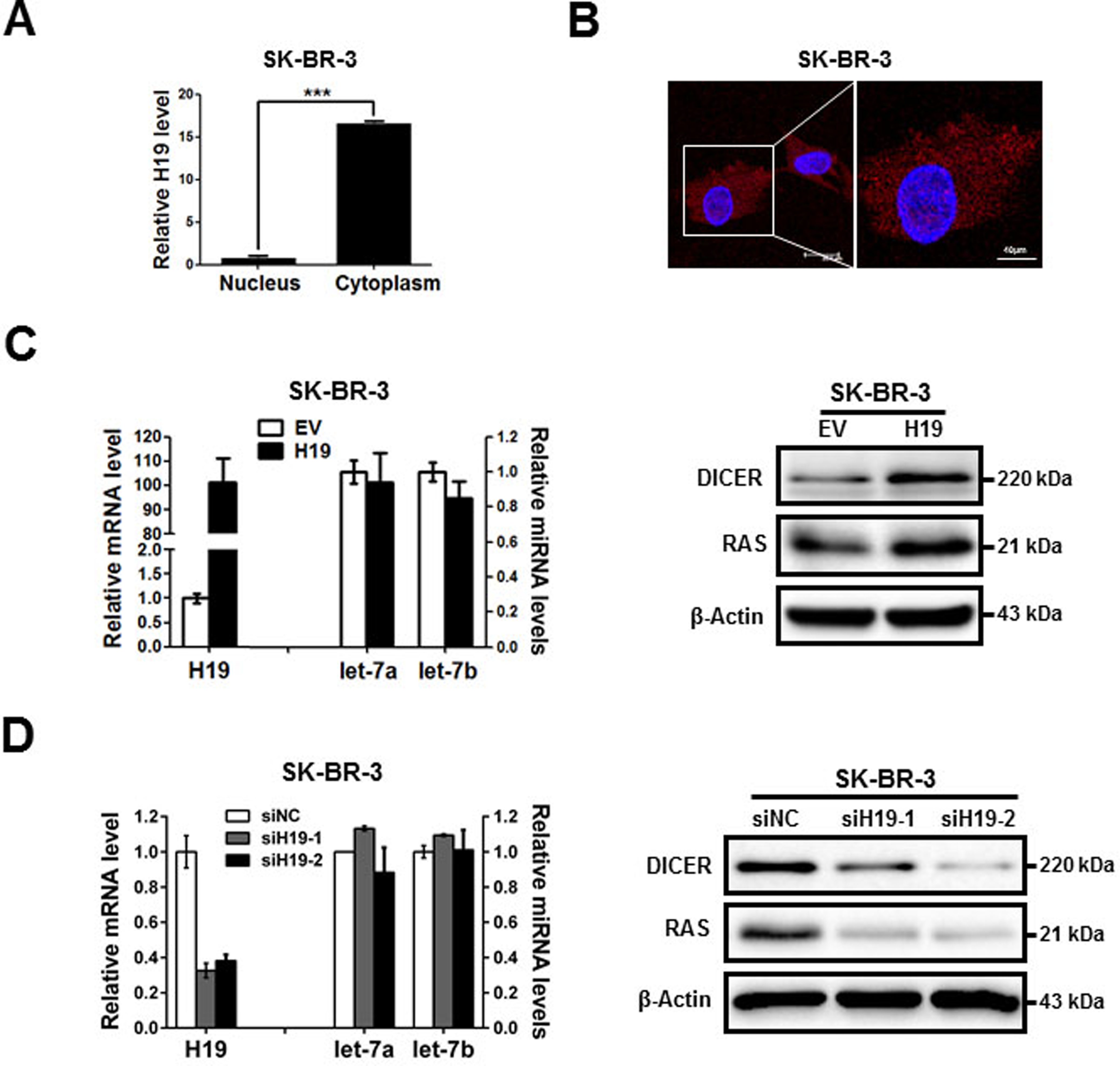

Supplement: Supplementary Figure 4 [file cddis2016438x7.tif]

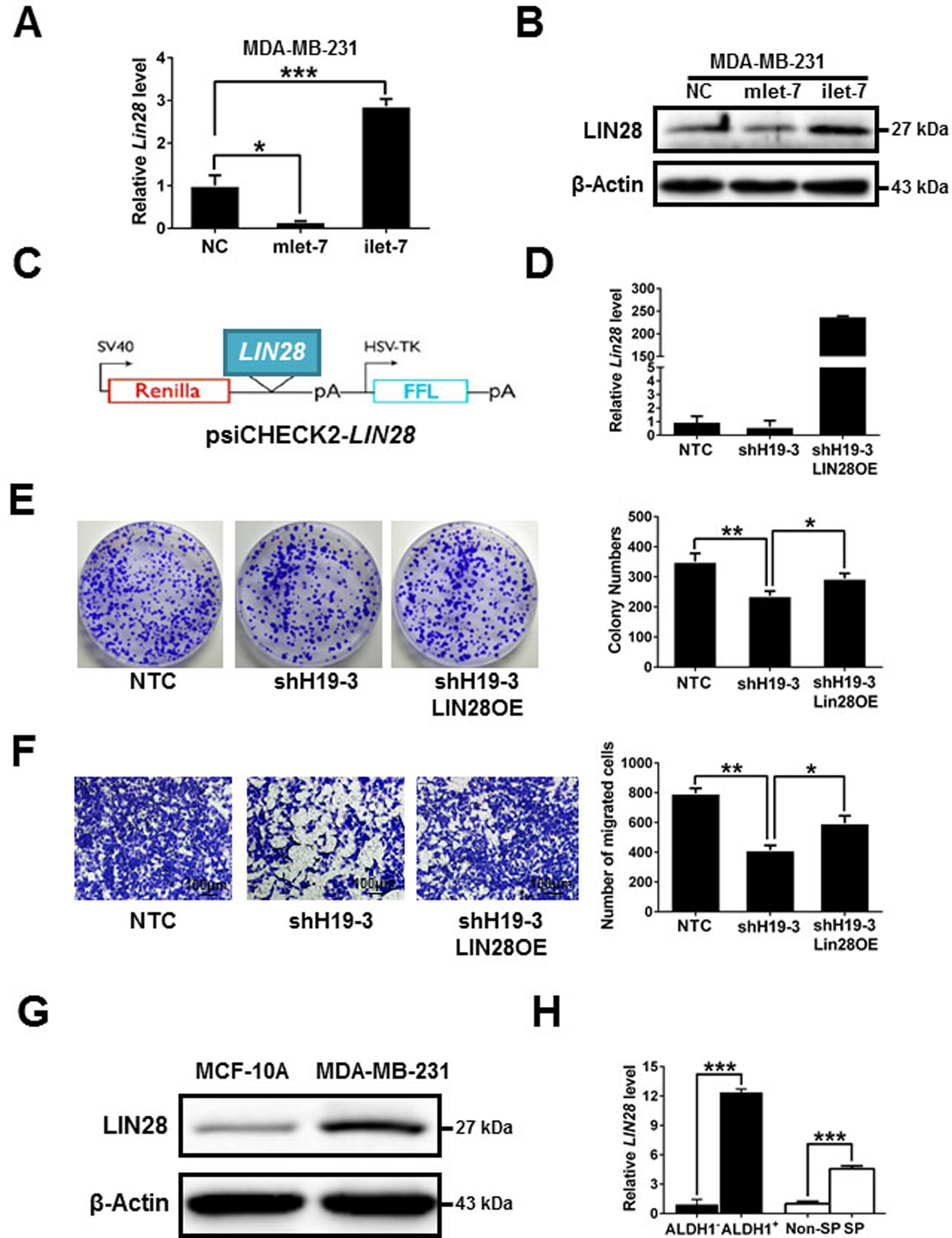

Supplement: Supplementary Figure 5 [file cddis2016438x8.tif]

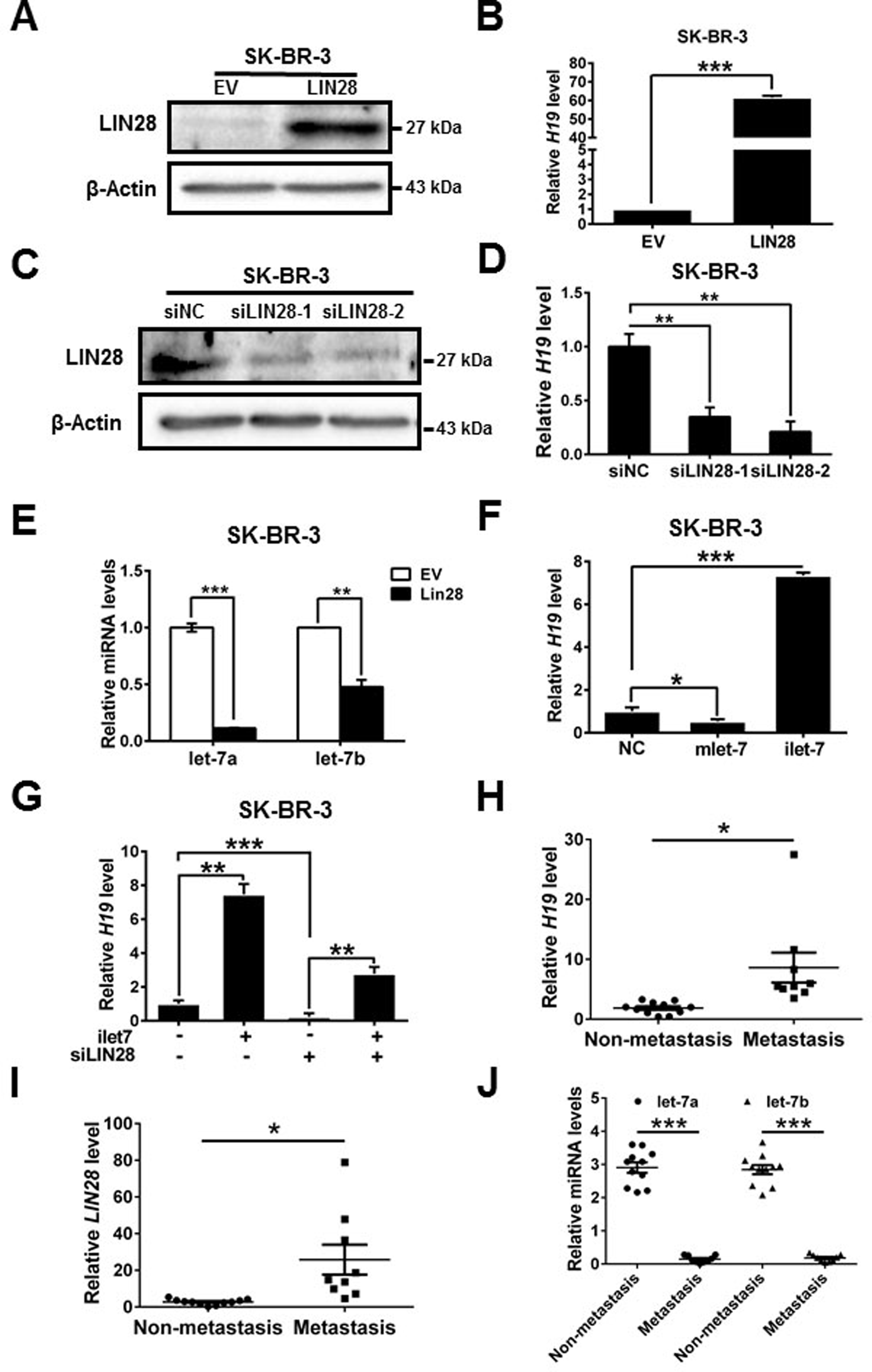

Supplement: Supplementary Figure 6 [file cddis2016438x9.tif]
