# Supplementary material for: H19/let-7/LIN28 reciprocal negative regulatory circuit promotes breast cancer stem cell maintenance
Source: Cell Death Dis. 2017 Jan 19;8(1):e2569–. doi: 10.1038/cddis.2016.438 (PMC5386357; doi:10.1038/cddis.2016.438)
Supplement: Supplementary Figure 7 [file cddis2016438x10.pdf]

The primers used in quantitative PCR are shown

Genes	Forward Primer 5′ >3′	Reverse Primer 5′ >3′
ACTB	ATCAAGATCATTGCTCCTCCTGAG	CTGCTTGCTGATCCACATCTG
H19	GCACCTTGGACATCTGGAGT	TTCTTTCCAGCCCTAGCTCA
POU5F1	GTGGAGGAAGCTGACAACAA	GCCGGTTACAGAACCACACT
SOX2	GGTTACCTCTTCCTCCCACTCC	CCCTCCCATTTCCCTCGTTT
NANOG	ACCTATGCCTGTGATTTGTGG	AGTGGGTTGTTTGCCTTTGG
LIN28	CGGGCATCTGTAAGTGGTTC	CAGACCCTTGGCTGACTTCT
ALDH1	ATCAAAGAAGCTGCCGGGAA	GCATTGTCCAAGTCGGCATC
XIST	TACCACTCTGGCCACTACGA	CCCAGCAGTGGTCAGTCATT
**mouse-*H19***	AATGGTGCTACCCAGCTCATG	TCAGAACGAGACGGACTTAAAGAA
**mouse-*ACTB***	GTGGGCCGCTCTAGGCACCAA	CTCTTTGATGTCACGCACGATTTC

RNA probe sequences for *H19*

Name	Forward Primer 5′ >3′	Reverse Primer 5′ >3′
***H19* probe**	GGGTGGGGGGTAACGGGGGA	TCCCTGTCTGACCCAGGCCTG

Primer sequences for psiCHECK2-*LIN28*

Name	Forward Primer 5′ >3′	Reverse Primer 5′ >3′
**psiCHECK2-LIN28**	CAGGTCTTCTTCATATGTTCTTTC	TGGAAAAGGTTACAAGAAGCTGA

Primer sequences for *H19* and *LIN28*

**Name**	**Forward Primer 5′ >3′**	**Reverse Primer 5′ >3′**
H19	AGCAGGGTGAGGGAGGGGGTG	AGCAGGGTGAGGGAGGGGGTG
LIN28	AGCAGGGTGAGGGAGGGGGTG	AGCAGGGTGAGGGAGGGGGTG

shRNA sequences

**Name**	**5′ >3′**
**shH19-1**	GAGTTAGCAAAGGTGACATCT
**shH19-2**	GACGTGACAAGCAGGACATGA
**shH19-3**	GCACTACCTGACTCAGGAATC
**shH19-4**	GACCTCATCAGCCCAACATCA
**NTC**	TTCTCCGAACGTGTCACGTTTC

siRNA sequences

**Name**	**Sense 5′ >3′**	**Anti-sense 5′ >3′**
**siH19-1**	CCCGUCCCUUCUGAAUUUATT	UAAAUUCAGAAGGGACGGGTT
**siH19-2**	GCGGGUCUGUUUCUUUACUTT	AGUAAAGAAACAGACCCGCTT
**siLIN28-1**	GGAUGUCUUUGUGCACCAGTT	CUGGUGCACAAAGACAUCCTT
**siLIN28-2**	GGUUCACACCAUCACCCUUTT	AAGGGUGAUGGUGUGAACCTT
**shNC**	UUCUCCGAACGUGUCACGUTT	ACGUGACACGUUCGGAGAATT

microRNA mimics sequences

Name	Sense 5′ >3′	Anti-sense 5′ >3′
let-7a	UUGUACUACACAAAAGUACUG	CUAUACAACCUACUACCUCAUU
let-7b	UGAGGUAGUAGGUUGUGUGGUU	CCACACAACCUACUACCUCAUU

microRNA inhibitors sequences

Name	5′ >3′
let-7a	AACUAUACAACCUACUACCUCA
let-7b	AACCACACAACCUACUACCUCA
